# SAAL1, a novel oncogene, is associated with prognosis and immunotherapy in multiple types of cancer

**DOI:** 10.18632/aging.204224

**Published:** 2022-08-13

**Authors:** Wei Yang, Bing Han, Yecheng Chen, Feng Geng

**Affiliations:** 1 Department of Pulmonary and Critical Care Medicine, The First Hospital of China Medical University, Shenyang 110001, Liaoning, P.R. China; 2 Department of Pulmonary and Critical Care Medicine, General Hospital of Northern Theatre Command, Shenyang 110001, Liaoning, P.R. China

**Keywords:** pan-cancer, SAAL1, prognostic and therapeutic biomarker, malignant phenotypes, PD-L1

## Abstract

Serum amyloid A-like 1 (SAAL1) was recently identified as a novel oncogene in hepatocellular carcinoma (HCC). To explore the potential role of SAAL1 in other cancers, we conducted a pan-cancer analysis of SAAL1 expression and its association with tumor microenvironment (TME) immunological profiles, sensitivity to chemotherapy agents, response to immunotherapy, and patient prognosis. SAAL1 was overexpressed in most malignant tumors in association with poor prognosis. Moreover, its expression was positively correlated with TME-relevant immune and mismatch signatures, immunostimulatory infiltrating cells (CD4^+^ memory T cells, activated NK cells, M1 macrophages, and cytotoxic CD8^+^ T cells), microsatellite instability (MSI), tumor mutational burden (TMB), neoantigen load, and immune checkpoint markers (PD-L1, LAG-3 and CTLA-4) in multiple cancers. SAAL1 overexpression was also associated with immunotherapy response and overall survival (OS) in bladder cancer (BLCA) patients who had received anti-PD-L1 treatment. Gene set enrichment analysis (GSEA) further showed significant enrichment of SAAL1 in immune cell signaling, cell cycle, and cell adhesion pathways. Moreover, we detected tumor-specific correlations between SAAL1 expression and either chemoresistance or sensitivity to common chemotherapeutics. Lastly, we showed that SAAL1 silencing suppresses both malignant phenotype and expression of PD-L1 in lung cancer A549 cells *in vitro*. These findings suggest that SAAL1 contributes to tumorigenesis and antitumor immunity mechanisms in different cancer types, and may thus serve as both a prognostic biomarker and potential target for cancer immunotherapy.

## INTRODUCTION

Worldwide, cancer ranks as a leading cause of death and disability. According to recent estimates, lung cancer is the leading cause of cancer-related death (1.8 million deaths; 18%), followed by colorectal (9.4%), liver (8.3%), stomach (7.7%), and female breast (6.9%) cancers [1]. In recent years, and overcoming disappointing results obtained over many decades, immunotherapeutic treatments have been clinically validated for the treatment of many cancers [2]. Among several immunotherapeutic modalities, antibody-based immune checkpoint blockade (ICB) aims at extending the antitumor activity of T cells by blocking the interaction of inhibitory receptors expressed on the surface of immune cells with one or more specific ligands typically overexpressed in tumor cells. The main targets for ICB treatments are cytotoxic T-lymphocyte-associated antigen 4 (CTLA-4), lymphocyte-activation gene 3 (LAG-3), and programmed cell death 1(PD-1) or its ligand PD-L1 [3, 4].

Although ICB is generally well tolerated, about 10% of its recipients develop serious autoimmune effects that require specific management [5]. Therefore, the development of predictive biomarkers for ICB response is needed to optimize patient benefit, minimize the risk of toxicities, and guide combination approaches [6]. Of note, although high PD-L1 expression on tumor cells was associated with response to anti-PD-1 therapies in various malignancies, patients with PD-L1-negative tumors by immunohistochemistry can still achieve clinical benefit with anti-PD-1 or anti-PD-L1 therapies [7, 8]. Current evidence suggests that specific biomarkers and gene sets related to the tumor microenvironment (TME), e.g., tumor-infiltrating immune cells, as well as tumoral features such as microsatellite instability (MSI), tumor mutational burden (TMB), neoantigen load, and expression of immune checkpoint-related genes may serve as predictive biomarkers for checkpoint inhibitor immunotherapy [9–11]. However, at present, detection of these biomarkers is generally expensive and often difficult. Therefore, there is a pressing need to identify more convenient and economical methods to predict tumor response to immunotherapy so that more patients can benefit from it.

The serum amyloid A-like 1 (SAAL1) gene is localized to chromosome 11p15.1. It consists of 12 exons interrupted by 11 introns [12], and encodes a protein with 474 amino acids and a molecular weight of about 54 kDa, which is mainly distributed in the nucleus [13]. SAAL1 belongs to the serum amyloid A (SAA) superfamily of proteins, which are, along with C-reactive protein, the major positive acute phase proteins in humans [12]. A proteomics study identified human SAAL1 as a phosphoprotein upregulated in synovial fibroblasts within rheumatoid joints, and its corresponding gene sequence was shown to be conserved from zebrafish to humans [13, 14]. Interestingly, a recent study identified SAAL1 as a novel oncogene in HCC [15]. Since the potential involvement of SAAL1 in the pathogenesis and progression of other cancer types, as well as its significance in cancer immunotherapy, remain unexplored, in this study we conducted a pan-cancer analysis of SAAL1 expression and its association with TME immunological characteristics, sensitivity to chemotherapy agents, response to anti-PD-L1 immunotherapy, and patient prognosis.

## RESULTS

### Expression of SAAL1 in different types of cancer

**Figure 1:**
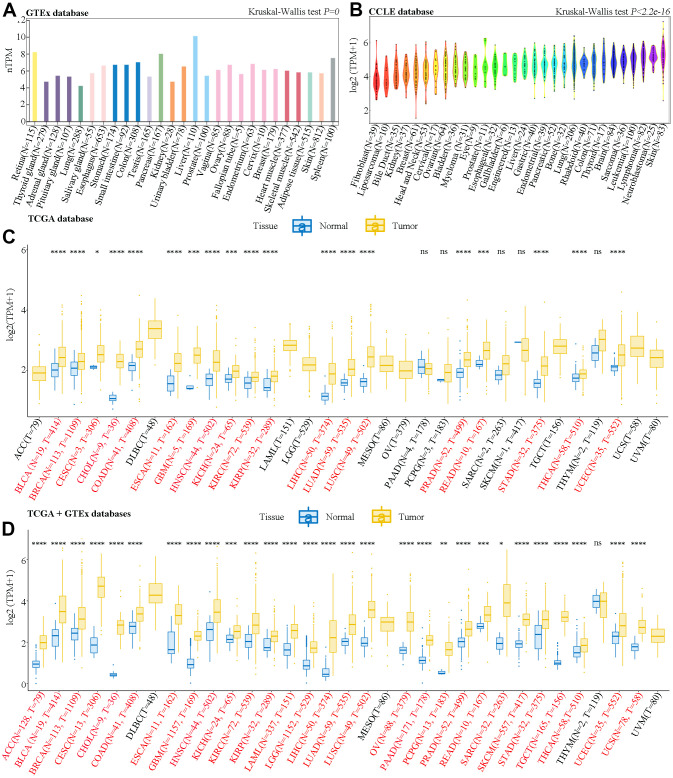
**Analysis of SAAL1 mRNA expression.** Expression levels of SAAL1 in (**A**) normal tissues, (**B**) tumor cell lines, (**C**) tumor tissues and paired adjacent noncancerous tissues in TCGA datasets, and (**D**) normal and tumor tissue samples combined, respectively, from GTEx and TCGA databases. *P<0.05; **P<0.01; ***P<0.001; ****P<0.0001; ns, not significant.

Datasets obtained from the Genotype-Tissue Expression (GTEx) database indicated that SAAL1 was lowly expressed in almost all human normal tissues, with liver and lung showing the highest and lowest expression levels, respectively (Figure 1A). In turn, analysis of the Cancer Cell Line Encyclopedia (CCLE) datasets showed that SAAL1 was expressed in most cancer cell lines, and predominantly overexpressed in skin cancer cell lines (Figure 1B).

SAAL1 expression in cancer and paired normal adjacent tissue samples was then analyzed in The Cancer Genome Atlas (TCGA) datasets. Results showed that SAAL1 was significantly upregulated in BLCA, BRCA, CESC, CHOL, COAD, ESCA, GBM, HNSC, KICH, KIRC, KIRP, LIHC, LUAD, LUSC, PRAD, READ, STAD, THCA, and UCEC tumor samples (Figure 1C). Moreover, after combining normal tissue data from the GTEx database with tumor tissue data from TCGA, SAAL1 was found to be significantly upregulated in ACC, BLCA, BRCA, CESC, CHOL, COAD, ESCA, GBM, HNSC, KICH, KIRC, KIRP, LAML, LGG, LIHC, LUAD, LUSC, OV, PAAD, PCPG, PRAD, READ, SARC, SKCM, STAD, TGCT, THCA, UCEC, and UCS (Figure 1D).

**Figure 2:**
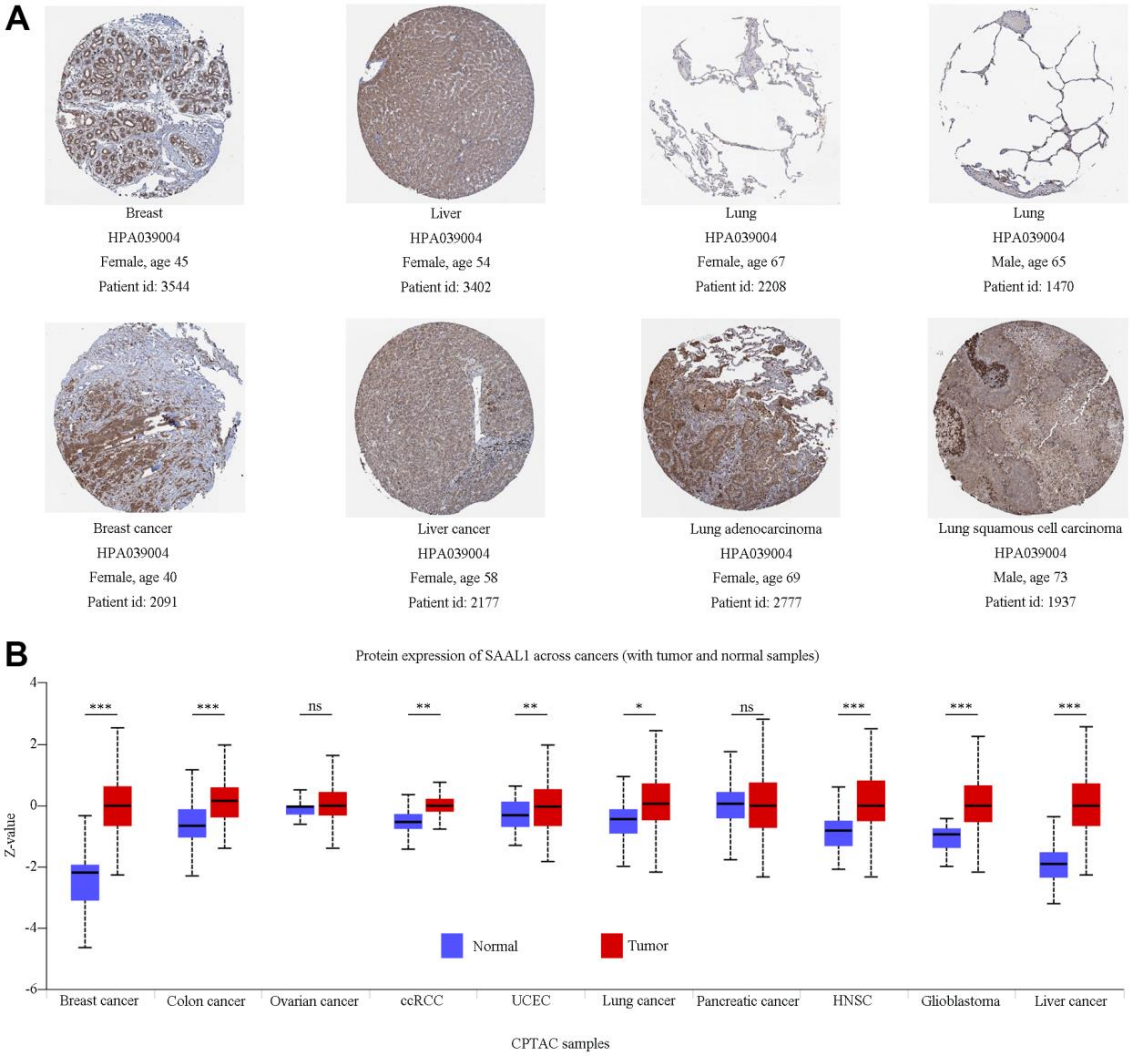
**Analysis of SAAL1 protein expression.** (**A**) Representative immunohistochemical staining for SAAL1 in normal and tumoral breast, liver, and lung tissues (images obtained from HPA). (**B**) SAAL1 protein expression levels in normal and tumor tissues. *P<0.05; **P<0.01; ***P<0.001; ns, not significant.

In addition, immunohistochemistry data from the Human Protein Atlas (HPA) indicated that SAAL1 was expressed at medium and high levels in normal breast and liver tissues, respectively, and these expression patterns did not differ significantly from those detected in the corresponding tumors (Figure 2A). In contrast, SAAL1 protein was expressed at low levels in normal lung tissues, but markedly upregulated in LUAD and LUSC tissues (Figure 2A). Moreover, in the National Cancer Institute’s Clinical Proteomic Tumor Analysis Consortium (CPTAC) database, SAAL1 protein was significantly upregulated in breast cancer, colon cancer, ccRCC, UCEC, lung cancer, HNSC, GBM, and liver cancer compared with corresponding normal tissues. In turn, there was no significant difference in SAAL1 protein expression levels between ovarian and pancreatic cancer tissues and their normal counterparts (Figure 2B).

**Figure 3:**
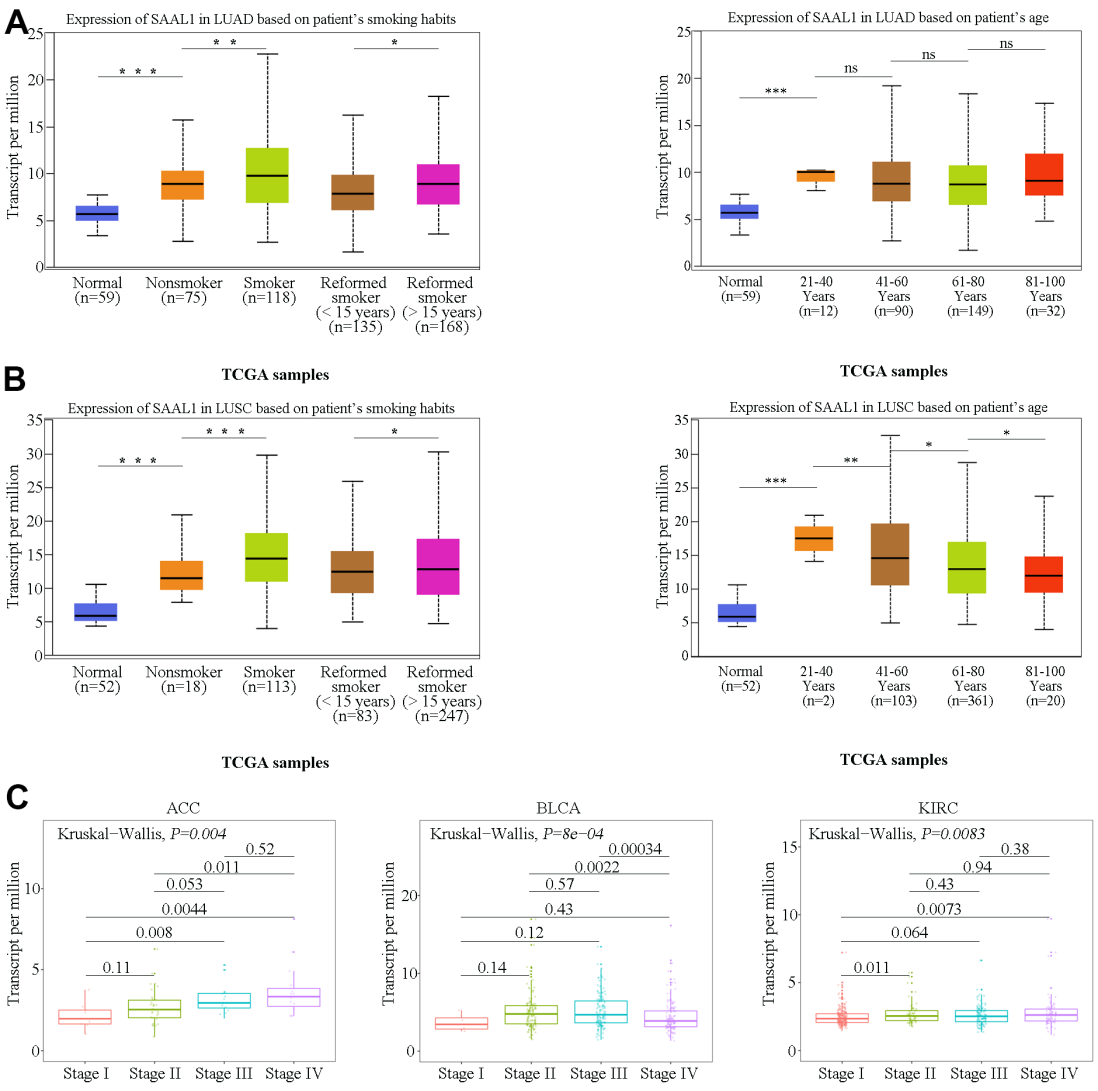
**Correlation analysis of SAAL1 expression and clinical parameters.** SAAL1 expression in (**A**) LUAD and (**B**) LUSC stratified according to patients’ smoking history and age. (**C**) SAAL1 expression as a function of tumor stage. *P<0.05; **P<0.01; ***P<0.001; ns, not significant.

Clinical correlation analysis using TCGA data indicated that SAAL1 expression was significantly higher in LUAD and LUSC patients with a history of smoking than in those without (Figure 3A, 3B). In addition, SAAL1 expression declined with age in LUSC, but not in LUAD (Figure 3A, 3B). We further assessed the expression of SAAL1 in different cancer stages and found stage-dependent increases in some tumors, including ACC, BLCA, and KIRC. However, in both BLCA and KIRC, the expression of SAAL1 began to decrease or remained constant when the tumors reached stage IV (Figure 3C).

### Survival analysis for SAAL1 expression

**Figure 4:**
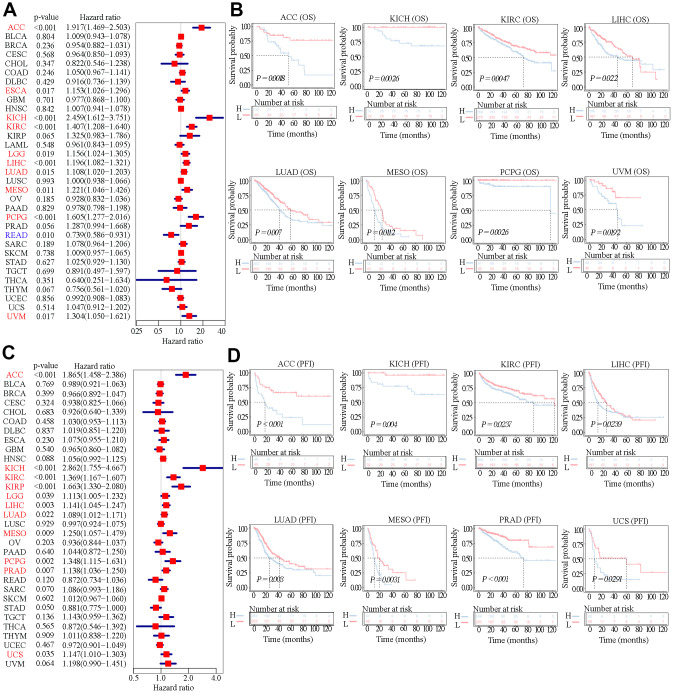
**Relationship between SAAL1 expression and OS and PFI.** (**A**) Forest plot of hazard ratios for OS (univariate survival analysis) in various cancer types. (**B**) Kaplan-Meier curves for OS derived from patients stratified according to SAAL1 gene expression. (**C**) Forest plot of hazard ratios for PFI. (**D**) Kaplan-Meier curves for PFI.

Since SAAL1 was highly expressed in most tumor types, the UCSC Xena database was used to examine the correlation between SAAL1 expression and clinical prognosis in different types of cancer. Analysis of overall survival (OS) using the Cox univariate shrinkage (uniCox) model indicated that SAAL1 was a risk factor for OS in ACC (HR=1.917, P<0.001), ESCA (HR=1.153, P=0.017), KICH (HR=2.459, P<0.001), KIRC (HR=1.407, P<0.001), LGG (HR=1.156, P=0.019), LIHC (HR=1.196, P<0.001), LUAD (HR=1.108, P=0.015), MESO (HR=1.221, P=0.011), PCPG (HR=1.605, P<0.001), and UVM (HR=1.304, P=0.017). Surprisingly, SAAL1 expression was associated with decreased risk in READ (HR= 0.739, P=0.010) (Figure 4A). On progression-free interval (PFI) analysis, SAAL1 was a risk factor in ACC (HR=1.865, P<0.001), KICH (HR=2.862, P<0.001), KIRC (HR=1.369, P<0.001), KIRP (HR=1.663, P<0.001), LGG (HR=1.113, P=0.039), LIHC (HR=1.141, P=0.003), LUAD (HR=1.089, P=0.022), MESO (HR=1.250, P=0.009), PCPG (HR=1.348, P=0.002), PRAD (HR=1.138, P=0.007), and UCS (HR=1.147, P=0.035) (Figure 4C).

In addition, Kaplan-Meier (KM) analysis of OS indicated that high SAAL1 expression predicted worse OS in patients with ACC, KICH, KIRC, LIHC, LUAD, MESO, PCPG, and UVM (Figure 4B). Meanwhile, KM curves for PFI showed that high SAAL1 expression predicted shorter PFI times in patients with ACC, KICH, KIRC, LIHC, LUAD, MESO, PRAD, and UCS (Figure 4D).

### Alterations and promoter methylation status of the SAAL1 gene in different types of cancer

**Figure 5:**
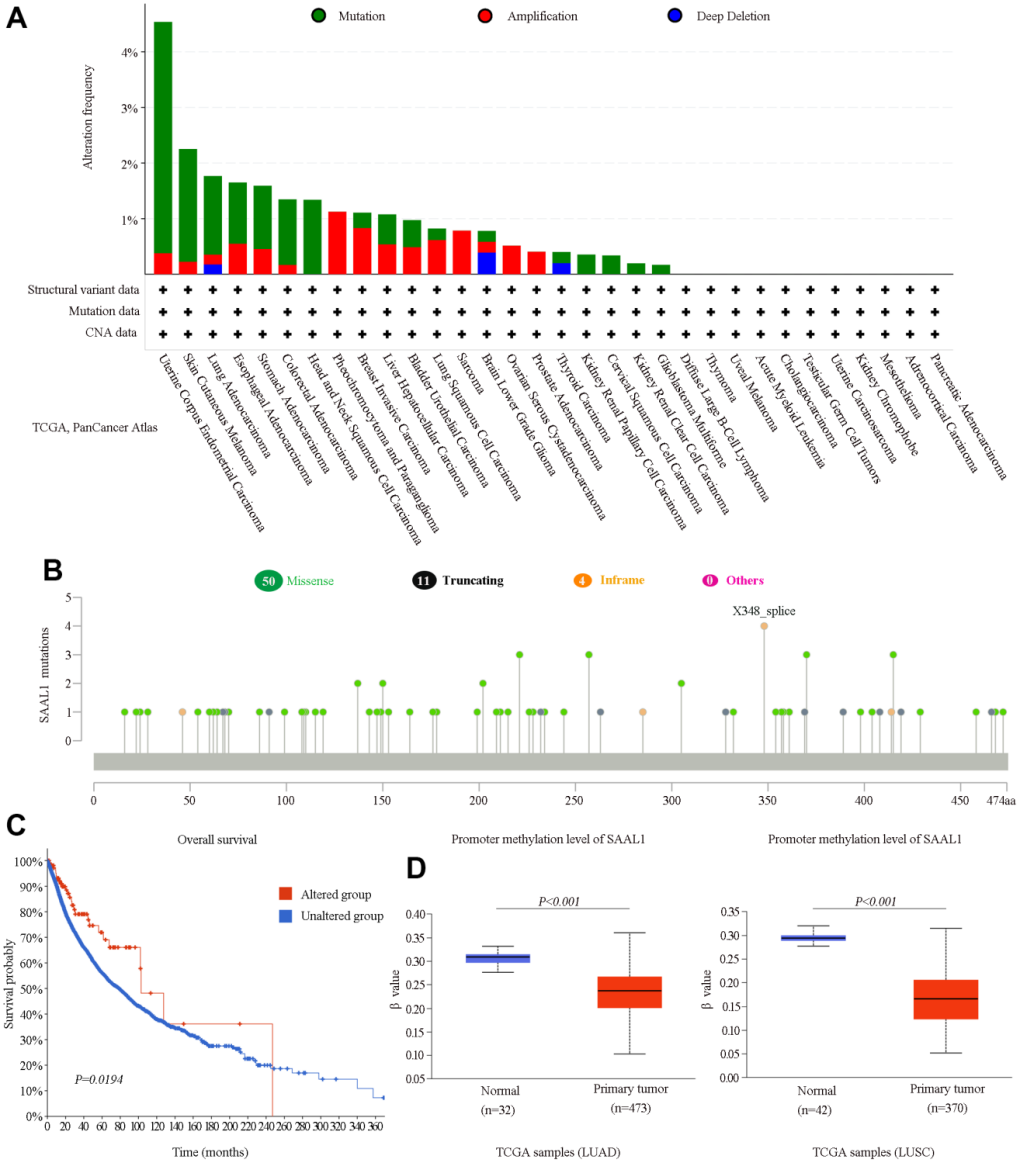
**Analysis of SAAL1 gene alterations and promoter methylation status in various cancer types.** (**A**) SAAL1 gene alteration types and frequencies in different cancer types. (**B**) Mutation types, sites, and frequencies (case numbers). (**C**) Survival curves for cancer patients with and without altered SAAL1. (**D**) SAAL1 promoter methylation status in LUAD and LUSC.

Based on data from the cBioPortal database, mutations were the most prevalent alterations in the SAAL1 gene among different cancers, with UCEC and SKCM showing in turn the highest mutation rates (>2%) (Figure 5A). In turn, missense mutations were the main type of mutations for the different cancers analyzed (Figure 5B). Moreover, pan-cancer KM analysis of OS indicated that patients with SAAL1 gene alterations had improved OS compared with those with unaltered SAAL1 (Figure 5C). Furthermore, as shown in Figure 5D, SAAL1 promoter methylation levels were significantly lower in LUAD and LUSC than in normal lung tissues.

### Correlation of SAAL1 expression with tumor-infiltrating immune cells and TME-relevant signatures

**Figure 6:**
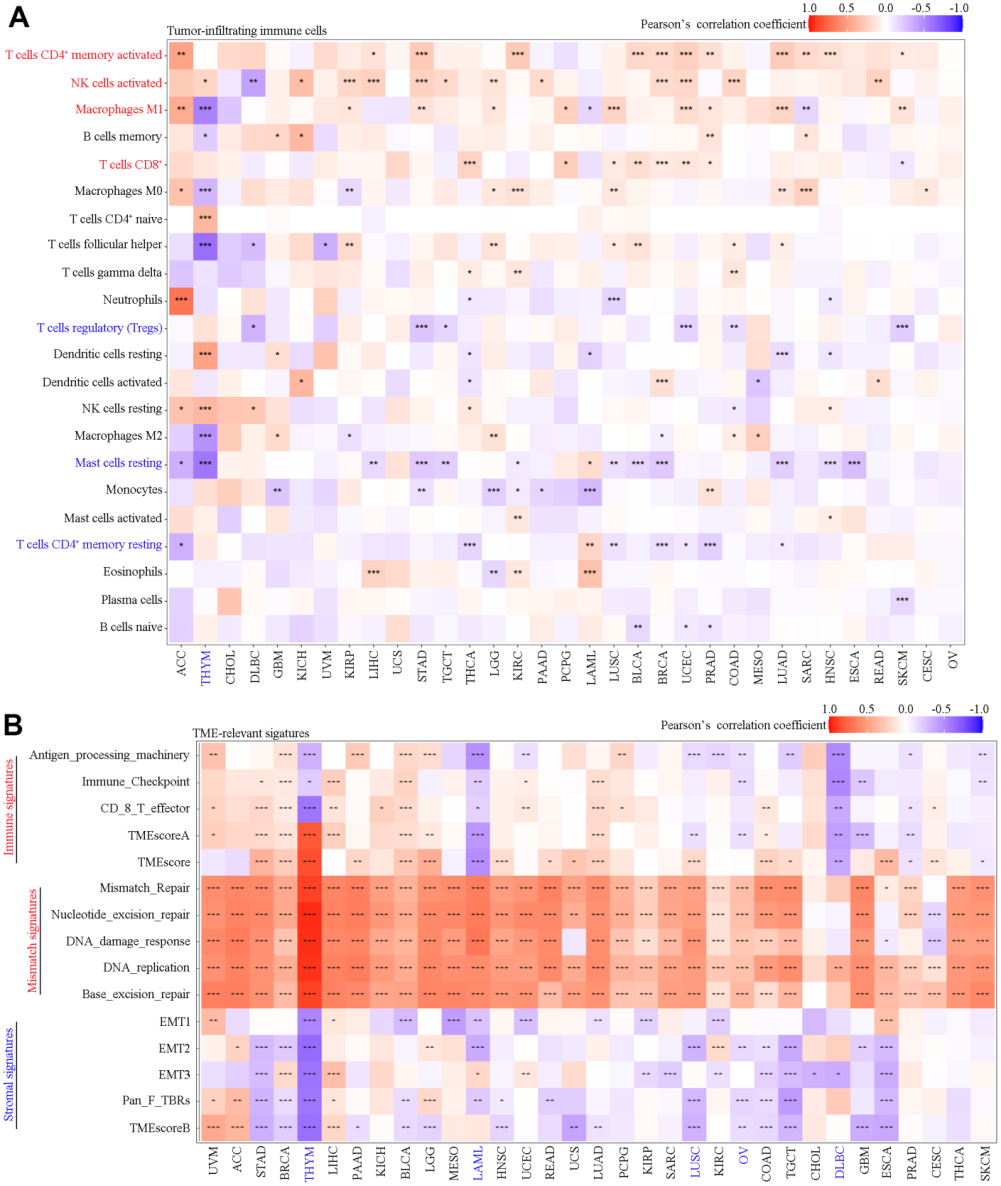
**Correlation analysis of SAAL1 expression with tumor-infiltrating immune cells and TME-relevant signatures in various cancer types.** (**A**) Tumor-infiltrating immune cells. (**B**) TME-relevant signatures. *P<0.05; **P<0.01; ***P<0.001.

Using the CIBERSORT algorithm, we found that in most cancer types SAAL1 expression was positively correlated with the frequency of favorable tumor-infiltrating immune cells (i.e., favoring anti-tumor immunity) such as activated CD4^+^ memory T cells, activated NK cells, M1 macrophages, and cytotoxic CD8^+^ T cells, and negatively correlated with unfavorable tumor-infiltrating immune cells (i.e., favoring suppression of anti-tumor immunity) such as regulatory T cells (Tregs), resting mast cells, and resting CD4^+^ memory T cells (Figure 6A).

As shown in Figure 6B, SAAL1 expression was positively correlated with immune signatures in most types of cancer, except for HYM, LAML, LUSC, OV, DLBC, GBM, PRAD, and SKCM. SAAL1 expression was also positively correlated with mismatch signatures in most types of cancer, except for CHOL, DLBC, and CESC. Lastly, SAAL1 expression was negatively correlated with stromal signatures in most types of cancer, except for UVM, ACC, LIHC, and LGG.

### Association of SAAL1 expression with immune-relevant genes

**Figure 7:**
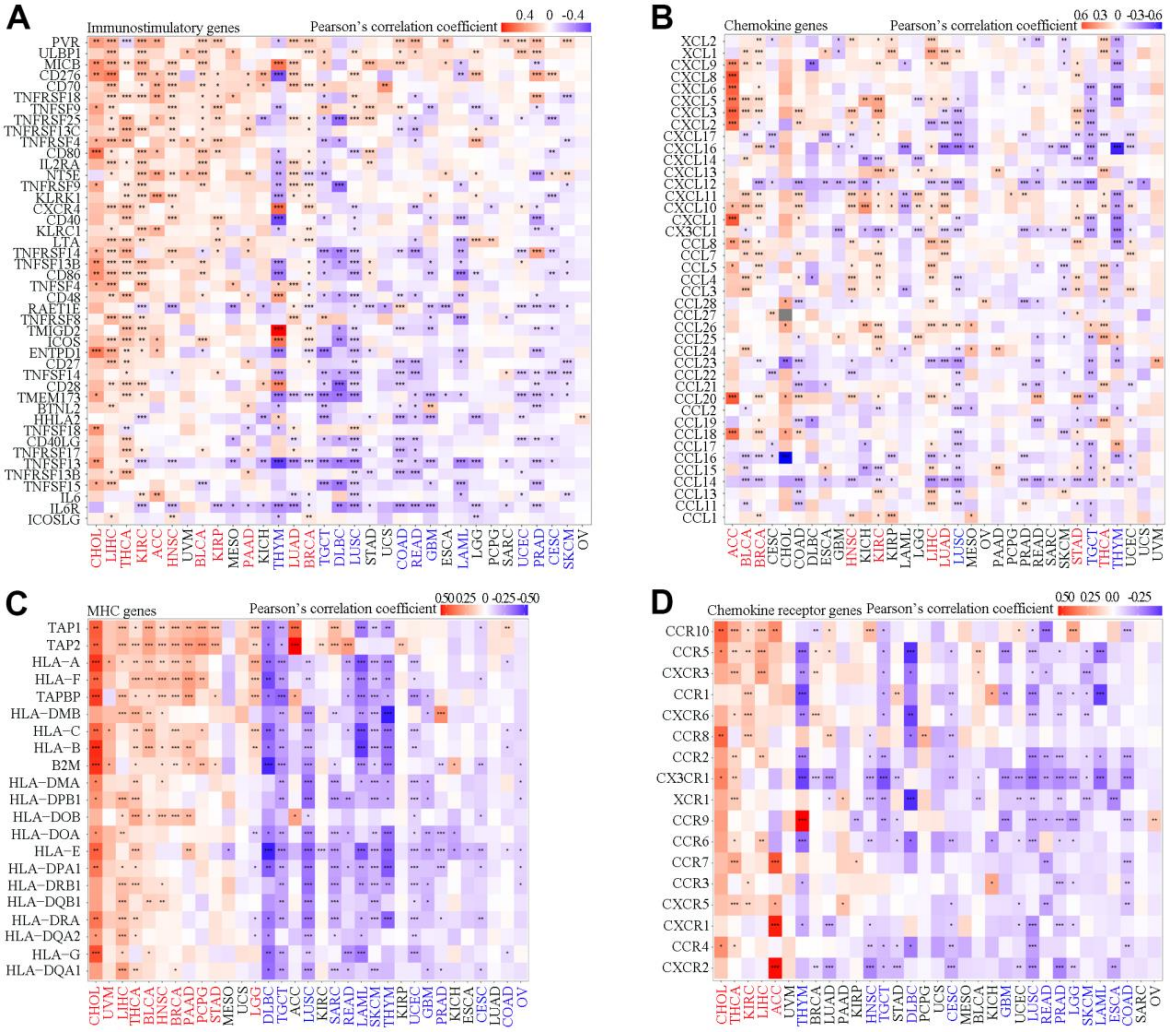
**Correlation analysis of SAAL1 expression and immune-relevant genes in various cancer types.** (**A**) Immunostimulatory genes. (**B**) Chemokine genes. (**C**) MHC genes. (**D**) Chemokine receptor genes. *P<0.05; **P<0.01; ***P<0.001.

SAAL1 expression correlated with that of several immune-relevant genes, albeit with different patterns in different tumor types. As shown in Figure 7A, SAAL1 was positively correlated with immunostimulatory genes in most types of cancers, including CHOL, LIHC, THCA, KIRC, ACC, HNSC, BLCA, KIRP, PAAD, LUAD, and BRCA. However, a negative correlation was found for THYM, TGCT, DLBC, LUSC, COAD, READ, GBM, LAML, UCEC, PRAD, CESC, and SKCM. For chemokine-coding genes, a positive correlation was found for ACC, BLCA, BRCA, HNSC, KIRC, LIHC, LUAD, STAD, and THCA, and a negative correlation was detected for LUSC, TGCT, THYM, and DLBC (Figure 7B). For MHC genes, a positive correlation was found for CHOL, UVM, LIHC, THCA, BLCA, HNSC, BRCA, PAAD, PCPG, STAD, and LGG, whereas a negative correlation was noted for DLBC, TGCT, LUSC, SARC, READ, LAML, SKCM, THYM, UCEC, GBM, PRAD, CESC, COAD, and OV (Figure 7C). Regarding the association between SAAL1 and chemokine receptor genes, a positive correlation was observed for CHOL, THCA, KIRC, LIHC, and ACC, whereas this correlation was negative for THYM, HNSC, TGCT, DLBC, CESC, GBM, LUSC, READ, PRAD, LGG, SKCM, LAML, ESCA, and COAD (Figure 7D). In summary, SAAL1 expression showed an especially evident positive association with immune-relevant genes in CHOL, THCA, LIHC, and STAD, whereas a mostly negative correlation was instead detected for THYM, TGCT, DLBC, and LUSC.

### Functional enrichment analysis of SAAL1 in different types of cancer

**Figure 8:**
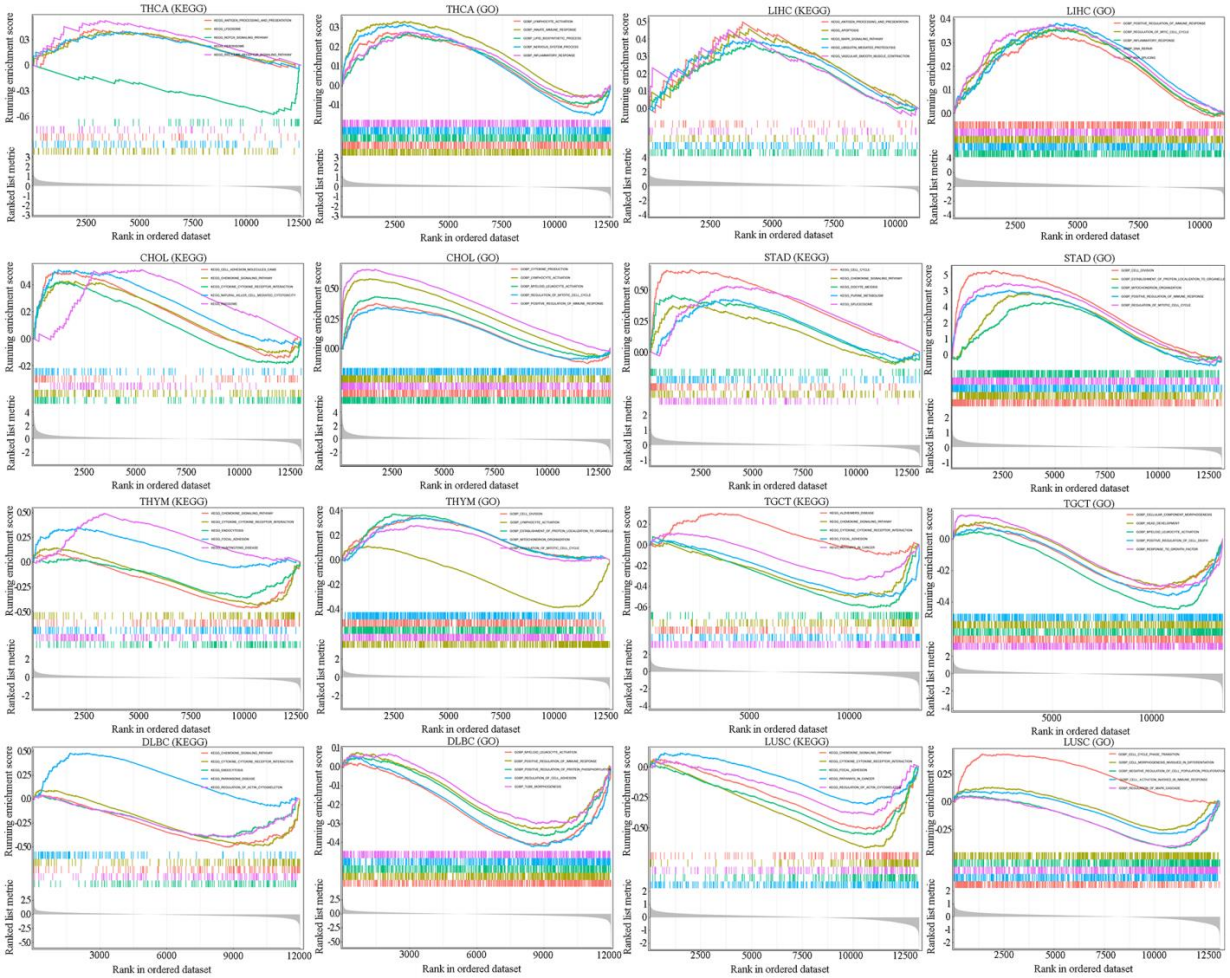
**GSEA for SAAL1-enriched KEGG pathways and GO terms in tumors (THCA, LIHC, CHOL, STAD, THYM, TGCT, DLBC and LUSC) with high and low SAAL1 expression.** The upward trend of the enrichment score (ES) lines denote pathway enrichment in the high SAAL1 expression group, and the downward trend of the ES lines indicate pathway enrichment in the low SAAL1 expression group. The top five items with the highest correlation with SAAL1 expression were analyzed by KEGG and GO enrichment analysis.

To explore the potential mechanisms by which SAAL1 may contribute to carcinogenesis, gene set enrichment analysis (GSEA) was performed in TCGA cohorts to identify functionally enriched Kyoto Encyclopedia of Genes and Genomes (KEGG) pathways and Gene Ontology (GO) terms related to immune-relevant pathways in high and low SAAL1-expressing cancers (Figure 8). KEGG enrichment analysis showed that high expression of SAAL1 was mainly associated with KEGG-ANTIGEN PROCESSING AND PRESENTATION (THCA and LIHC), KEGG-CHEMOKINE SIGNALING PATHWAY (CHOL and BRCA), KEGG-CYTOKINE CYTOKINE RECEPTOR INTERACTION (CHOL), and KEGG-NATURAL KILLER CELL MEDIATED CYTOTOXICITY (CHOL). In turn, low expression of SAAL1 was mainly associated with KEGG-CHEMOKINE SIGNALING PATHWAY (THYM, TGCT, DLBC, and LUSC), and KEGG-CYTOKINE CYTOKINE RECEPTOR INTERACTION (THYM, TGCT, DLBC, and LUSC). Next, the association of tumors with high and low SAAL1 expression and immune-related processes was assessed through GO enrichment analysis. Results indicated that high expression of SAAL1 was mainly associated with GOBP-LYMPHOCYTE ACTIVATION (THCA and CHOL), GOBP-INNATE IMMUNE RESPONSE (THCA), GOBP-INFLAMMATORY RESPONSE (THCA and LIHC), and GOBP-POSITIVE REGULATION OF IMMUNE RESPONSE (LIHC, CHOL, and BRCA). In turn, low expression of SAAL1 was mainly associated with GOBP-LYMPHOCYTE ACTIVATION (THYM), GOBP-POSITIVE REGULATION OF IMMUNE RESPONSE (DLBC), and GOBP-CELL ACTIATION INVOVED IN IMMUNE RESPONSE (LUSC). In addition, we detected significant associations of SAAL1 expression with CELL CYCLE, REGULATION OF MAPK CASCADE, REGULATION OF PROTEIN PHOSPHORYLATION, and FOCAL ADHESION, among other GO terms.

### Association of SAAL1 with the immune checkpoint genes, MSI, TMB, and neoantigen load

**Figure 9:**
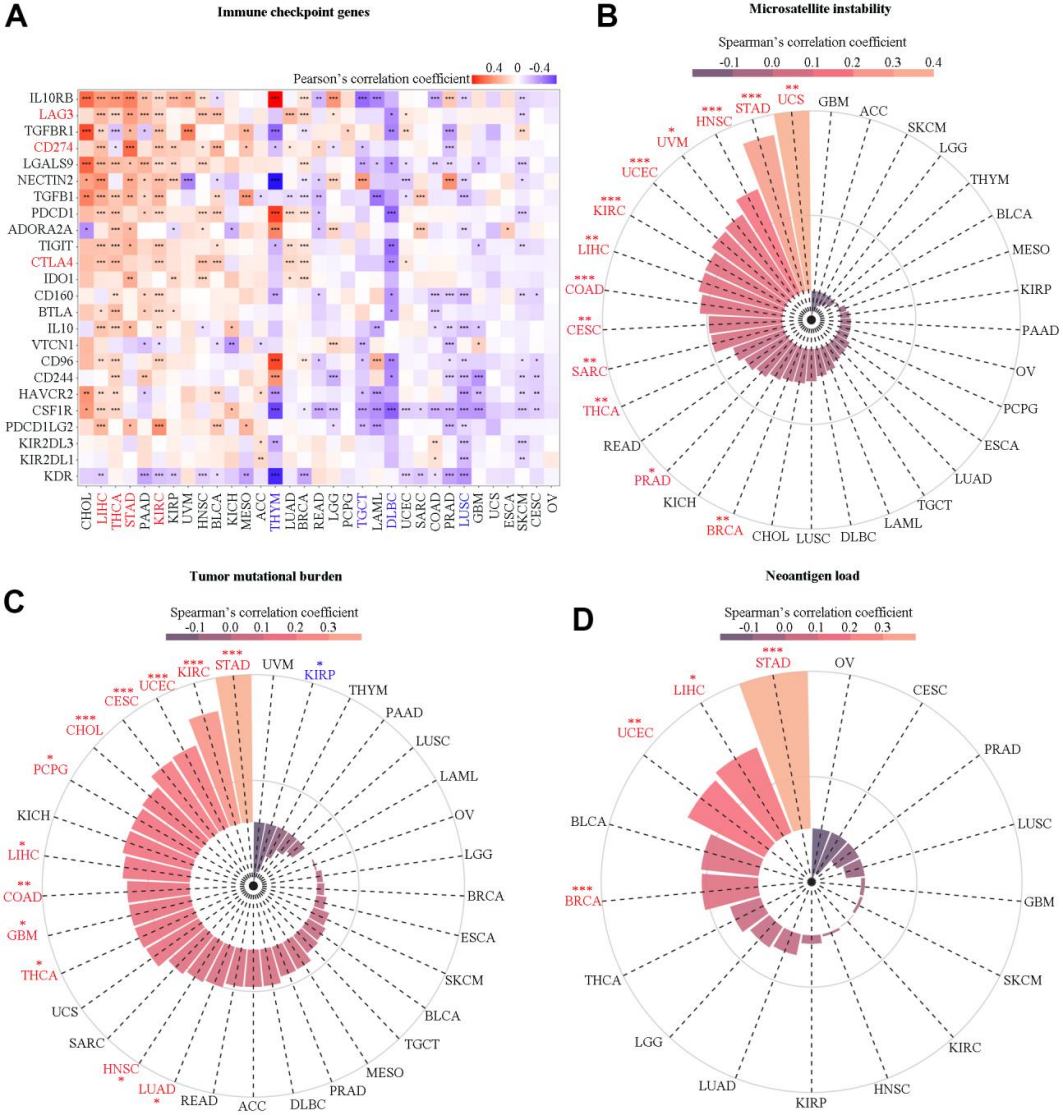
**Correlation analysis of SAAL1 with immune checkpoint genes, microsatellite instability, tumor mutational burden, and neoantigen load.** (**A**) Immune checkpoint genes. (**B**) Microsatellite instability. (**C**) Tumor mutational burden. (**D**) Neoantigen load. *P<0.05; **P<0.01; ***P<0.001.

Cancer prognosis is importantly determined by immunosurveillance efficacy, which is critically influenced by the expression of immune checkpoint-related genes, such as PD-L1, CTLA-4, and LAG-3, in cancer cells [16]. Hence, we examined the association between SAAL1 and immune checkpoint-related gene expression in different cancer types. As shown in Figure 9A, SAAL1 expression was positively correlated with immune checkpoint gene expression in numerous cancer types, including CHOL, LIHC, THCA, STAD, PAAD, KIRC, KIRP, HNSC, BLCA, LUAD, BRCA, and READ. In turn, a negative correlation between SAAL1 and immune checkpoint gene expression was detected in THYM, TGCT, LAML, DLBC, COAD, PRAD, LUSC, GBM, SKCM, and CESC. Furthermore, SAAL1 was positively correlated with CD274 (PD-L1) in most cancer types, including LIHC, STAD, KIRC, KIRP, HNSC, KICH, ACC, LUAD, and LGG. Regarding CTLA-4, a positive correlation with SAAL1 was observed in LIHC, THCA, KIRC, HNSC, BLCA, LUAD, BRCA, and UCEC. In addition, SAAL1 was positively correlated with LAG-3 in LIHC, THCA, STAD, PAAD, KIRC, KIRP, BLCA, KICH, LUAD, BRCA, LGG, and UCEC.

The expression of SAAL1 was significantly correlated with higher MSI in UCS, STAD, HNSC, UVM, UCEC, KIRC, LIHC, COAD, CESC, SARC, THCA, PRAD, and BRCA (Figure 9B). In addition, SAAL1 was positively correlated with TMB in STAD, KIRC, UCEC, CESC, CHOL, PCPG, LIHC, COAD, GBM, THCA, HNSC, and LUAD, and negatively correlated with TMB in KIRP (Figure 9C). As for neoantigen load, there was a positive correlation for SAAL1 in STAD, SKCM, UCEC, and BRCA (Figure 9D). Overall, these analyses indicated that SAAL1 expression was correlated with that of immune checkpoint genes, albeit with different patterns in different tumor types. Specifically, it is worth noting that SAAL1 was positively correlated with PD-L1, CTLA-4, and LAG-3 in most cancer types. Similarly, the association between SAAL1 expression and MSI, TMB, and neoantigen load was positive in most tumors.

### Cohort validation of the prognostic effect of SAAL1 on immunotherapy

**Figure 10:**
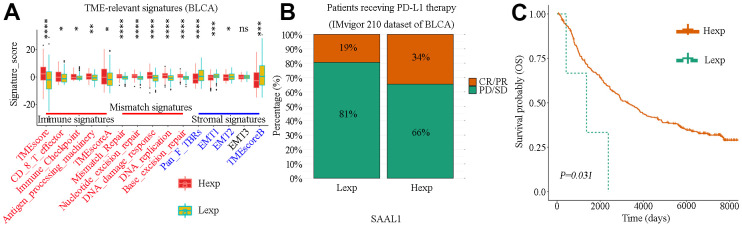
**Correlation analysis of SAAL1 expression and TME-relevant signatures and immune checkpoint blockade therapy in BLCA.** (**A**) Correlation analysis of SAAL1 expression and TME-relevant signatures. (**B**) Correlation between high and low SAAL1 expression and therapeutic response to PD-L1 blockade immunotherapy in BLCA patients. (**C**) SAAL1 expression-based Kaplan-Meier OS curves for BLCA patients who had received anti-PD-L1 immunotherapy. *P<0.05; **P<0.01; ***P<0.001; ns, not significant. Hexp, high SAAL1 expression; Lexp, low SAAL1 expression. Response: CR, complete response; PR, partial response. No response: PD, progressive disease; SD, stable disease.

The association of SAAL1 with TME-relevant signatures in BLCA was analyzed using the CIBERSORT algorithm. The results indicated that high expression of SAAL1 was significantly associated with immune signatures (TMEscoreA, Antigen-processing-machinery, Immune-Checkpoint, CD-8-T-effector, and TMEscore) and mismatch signatures (Base-excision-repair, DNA-replication, DNA-damage-response, Nucleotide-excision-repair, and Mismatch-Repair). On the contrary, low SAAL1 expression was significantly associated with stromal signatures (Pan-F-TBRs, EMT1, EMT2, and TMEscoreB) (Figure 10A). These results suggested that high expression of SAAL1 correlates with a favorable response to immunotherapy.

The success of PD-L1 blockade therapies in clinical trials has greatly advanced the interest in cancer immunotherapy [17]. Using data from the IMvigor210 study, the correlation between SAAL1 expression and therapeutic response to PD-L1 blockade immunotherapy was examined in BLCA patients. Results showed that the response rate in patients with high SAAL1 expression (73/216) was higher than in those with low SAAL1 expression (25/132) (34% vs 19%, χ^2^=8.939, P=0.003) (Figure 10B). Accordingly, KM analyses indicated that high SAAL1 expression was associated with improved OS in BLCA patients who had received PD-L1 immunotherapy (Figure 10C).

### SAAL1 drug sensitivity analysis in cancer cell lines

**Figure 11:**
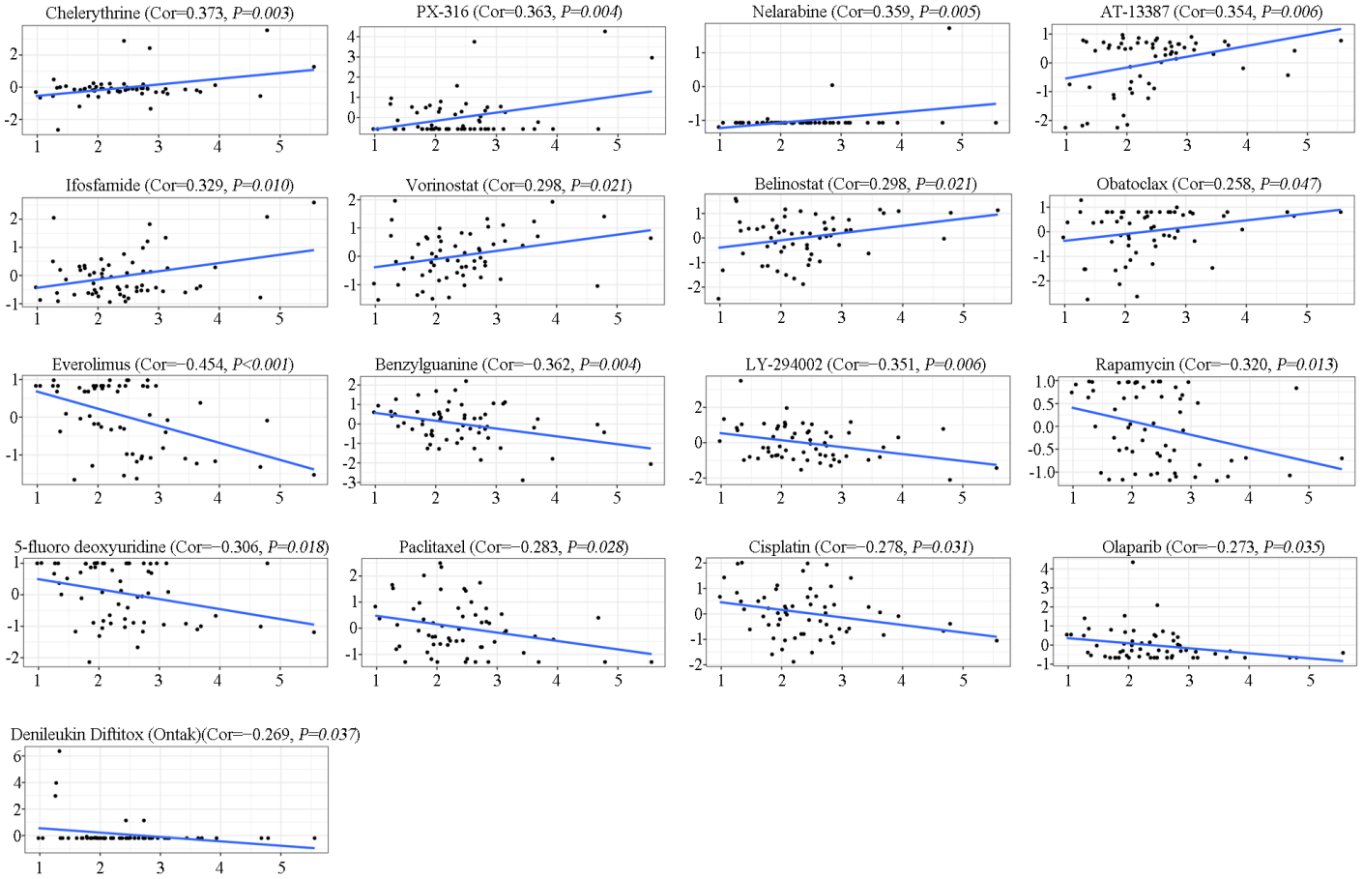
**Analysis of SAAL1 drug sensitivity.** The expression of SAAL1 was associated with the sensitivity of chelerythrine, PX-316, nelarabine, AT-13387, ifosfamide, vorinostat, belinostat, obatoclax, everolimus, benzylguanine, LY-294002, rapamycin, 5-fluoro deoxyuridine, paclitaxel, cisplatin, olaparib, and denileukin diftitox (Ontak). Cor, correlation coefficient.

To further investigate the potential correlation between SAAL1 expression and anticancer drug sensitivity, we accessed the CellMiner database to retrieve drug sensitivity and RNA-Seq information for the NCI-60 panel of cancer cell lines. Notably, SAAL1 expression was positively correlated with sensitivity to chelerythrine, PX-316, nelarabine, AT-13387, ifosfamide, vorinostat, belinostat, and obatoclax (Figure 11). Moreover, our results indicated that SAAL1 expression was negatively associated with sensitivity to everolimus, benzylguanine, LY-294002, rapamycin, 5-fluoro deoxyuridine, paclitaxel, cisplatin, olaparib, and denileukin diftitox (Ontak) (Figure 11). These data indicated that high SAAL1 expression might be associated with chemoresistance to common chemotherapeutic agents, including paclitaxel, cisplatin, and 5-fluoro deoxyuridine.

### SAAL1 depletion inhibits the malignant phenotype of lung cancer cells *in vitro*

**Figure 12:**
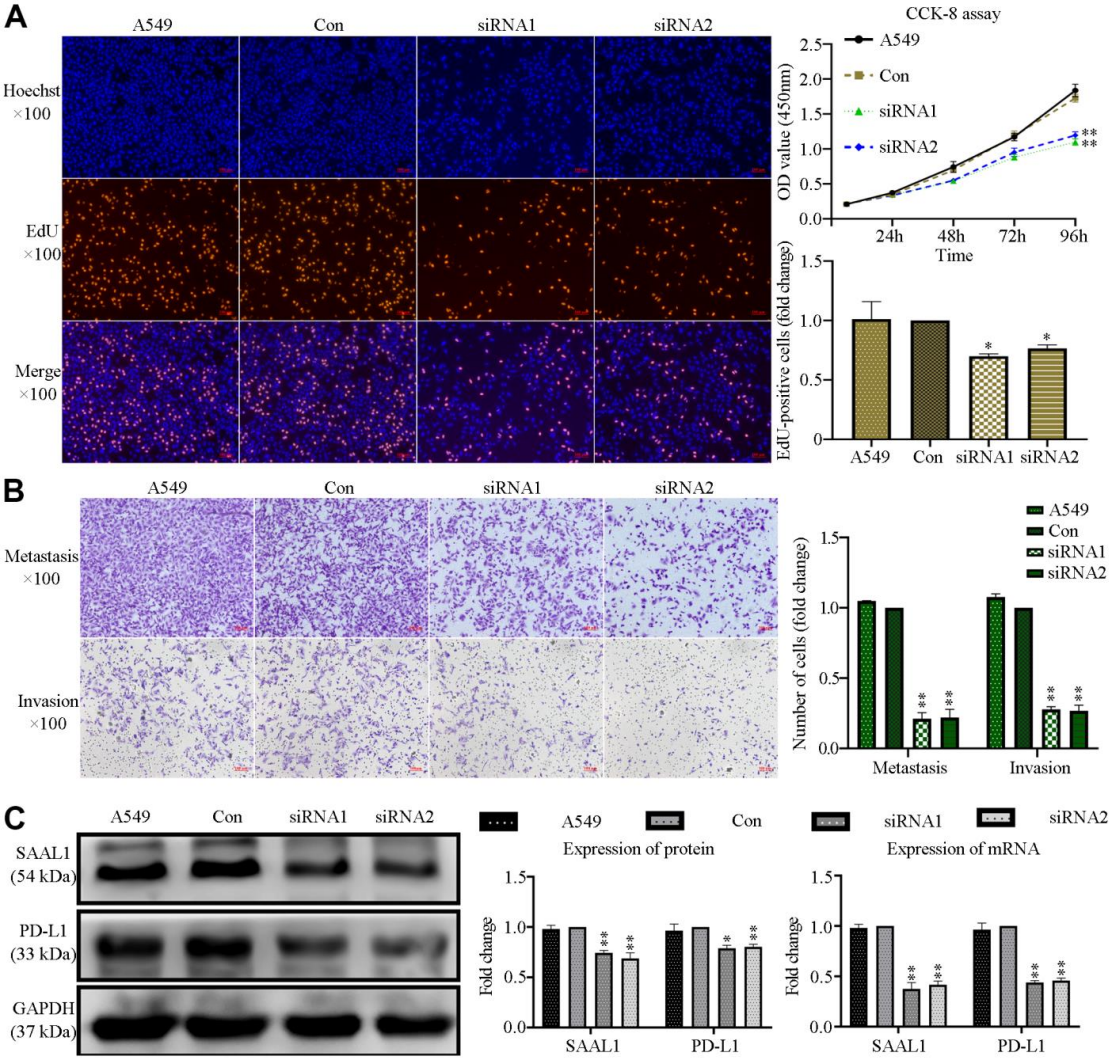
**SAAL1 silencing inhibits proliferation, migration, and invasion and downregulates PD-L1 expression in lung cancer cells.** A549 cells were transfected with control or SAAL1-targeting siRNAs (siRNA1 and siRNA2). (**A**) EdU and CCK-8 proliferation assay results. (**B**) Transwell migration and invasion assay results. (**C**) Western blotting and RT-qPCR analysis of SAAL1 and PD-L1 expression. GAPDH was used as the loading control. *P<0.05, **P<0.01 vs. Con group.

Since our analysis of CCLE datasets indicated that SAAL1 is overexpressed in lung cancer cell lines, we next explored the effect of SAAL1 on the proliferation, migration, and invasion of human lung A549 cancer cells. Results of EdU and CCK-8 assays indicated that siRNA-mediated depletion of SAAL1 significantly suppressed proliferation (Figure 12A), as well as migration and invasion (Figure 12B). Moreover, western blotting confirmed that SAAL1 was highly expressed in A549 cells, and its depletion significantly reduced both protein and mRNA levels of PD-L1 (Figure 12C).

## DISCUSSION

A recent study identified a novel oncogenic role for SAAL1, a ubiquitously expressed protein associated with cell cycle and liver inflammation, in HCC [15]. Since apart from the mentioned study the role of SAAL1 in other cancer types has not been clarified, we undertook a comprehensive pan-cancer analysis of SAAL1 expression and its association with tumor-related features.

Our data indicated that SAAL1 was expressed at low levels in almost all normal human tissues, which suggests an important role in embryonic development and/or essential cell processes. In turn, analysis of the GTEx database and the HPA repository indicated that the expression of SAAL1 was the highest in normal liver. These results are thus consistent with previous finding indicating that SAAL1 is mainly synthesized by hepatocytes, where it acts as an acute-phase protein during acute inflammation [12]. Analysis of CCLE datasets indicated that SAAL1 was expressed in most cancer cell lines, which suggested an important role in the development of multiple cancer types. In addition, most of the 19 cancer types examined in the present study exhibited higher SAAL1 expression than their corresponding normal tissues in TCGA datasets. Furthermore, when normal tissue data from GTEx database was combined with tumor tissue data from TCGA, SAAL1 mRNA was found to be significantly upregulated in 29/33 tumor types. In turn, assessment of proteomic data from the CPTAC database indicated that SAAL1 protein levels were significantly upregulated in eight types of cancer, compared with their normal counterparts. These results suggested that SAAL1 expression might be used as a diagnostic biomarker in selected tumors.

Smoking is closely associated with lung cancer. Our results showed that the expression of SAAL1 in patients with LUAD and LUSC who smoked was significantly higher than in those who did not smoke, suggesting that smoking may lead to high SAAL1 expression in LUAD and LUSC. Moreover, SAAL1 expression declined with age in LUSC, but not in LUAD. These results suggest that SAAL1 has heterogeneous biological functions in different types of tumors. We further observed that SAAL1 expression increased with tumor stage in ACC; this result suggested that SAAL1 may play an important role in the development of ACC.

According to uniCox analysis and KM curves of OS and PFI, high expression of SAAL1 was significantly associated with poor prognosis in ACC, ESCA, KICH, KIRC, LGG, LIHC, LUAD, MESO, PCPG, UVM, KIRP, PRAD, and UCS. Thus, SAAL1 was not only upregulated in multiple tumor types but also associated with their prognosis, which suggested that SAAL1 may represent a diagnostic biomarker and a prognostic indicator for some tumors. In this regard, because high SAAL1 levels are present in blood [12], and overexpression occurs in many malignant tumors, blood-based SAAL1 testing may constitute an easily available strategy for tumor diagnosis.

Subsequently, we accessed the cBioPortal database to examine the record of known alterations in the SAAL1 gene in different tumors. The results indicated that mutations were the main type of alterations in the SAAL1 gene in most cancer types evaluated. Therefore, it may be hypothesized that mutations in the SAAL1 gene mainly account for its abnormal expression in a variety of tumor tissues. Moreover, we found that cancer patients with SAAL1 gene alterations had better OS than those without alterations in this gene. DNA methylation is one of the most studied epigenetic modifications in mammals, and a major mechanism of transcriptional silencing. In tumor cells, DNA demethylation was shown to promote the expression of diverse oncogenes [18]. The present study showed that SAAL1 was overexpressed in LUAD and LUSC. Consistent with this observation, SAAL1 promoter methylation levels were significantly reduced in these tumor types. This suggested that promoter hypomethylation may be another reason for the high expression of SAAL1 in tumor tissues.

The TME shapes tumor development and progression through the interaction between tumor cells and surrounding stromal cells (e.g., fibroblasts, vascular and immune cells) that have distinct structural and immunological functions [19]. In the present study, SAAL1 expression correlated positively with favorable tumor-infiltrating immune cells such as activated CD4^+^ memory T cells, activated NK cells, M1 macrophages, and CD8^+^ T cells, and negatively with unfavorable tumor-infiltrating immune cells such as Tregs, resting mast cells, and resting CD4^+^ memory T cells. These results suggest that high expression of SAAL1 is conducive to the formation of an immune-inflamed phenotype in most human solid tumors. Noteworthy, many studies have suggested that this phenotype is most responsive to ICB therapies [20–22]. Moreover, with the exception of THYM, LAML, LUSC, OV, TGCT, and DLBC, in the other tumors analyzed the expression of SAAL1 was positively correlated with immune and mismatch signatures and negatively correlated with stromal signatures. These results suggest that ICB therapies might be more effective in high-SAAL1-expressing tumors. In addition, there was a marked correlation between the expression of SAAL1 and that of immune-relevant (e.g., immunostimulatory, chemokine-related, and MHC-related) genes in most tumors. Most notably, a positive correlation was noted for CHOL, THCA, LIHC, and STAD, whereas a negative correlation was observed for THYM, TGCT, DLBC, and LUSC. Supporting the observed correlations between tumoral SAAL1 expression and immune-relevant genes, functional enrichment analysis showed differential enrichment in various immune-relevant pathways for tumors with high and low SAAL1 expression. These findings strongly suggest that high and low SAAL1 expression promote and inhibit, respectively, antitumor immunity. However, the impact of SAAL1 on tumorigenesis may not be restricted to modulation of immunological responses, as significant associations between tumoral SAAL1 expression and various cellular processes, including cell cycle, MAPK signaling, regulatory protein phosphorylation, and cell adhesion, were further observed in the present study.

There was overall a significantly positive correlation between the expression of SAAL1 and that of PD-L1, CTLA-4, and LAG-3, three major immune checkpoint targets of immunotherapy. Moreover, SAAL1 expression was also positively correlated with MSI in 13 cancer types, with TMB in 12 cancer types, and with neoantigen load in 4 cancer types. Therefore, SAAL1 might be a pan-cancer biomarker of favorable prognosis for ICB therapy. We tested this hypothesis in a cohort of BLCA patients who had received immunotherapy. The results demonstrated that patients with high SAAL1 expression had higher response rates and longer OS after receiving PD-L1 blockade therapy. These findings would suggest that stimulation of SAAL1 expression in low-SAAL1-expressing tumors by pharmacological or biological agents may be effective to render them sensitive to immunotherapy. Thus, both cell-based and animal studies are needed to confirm these hypotheses in future studies.

A previous study showed that suppression of SAAL1 increased the chemosensitivity of HCC cells to sorafenib and foretinib treatments [15]. In our study, data from the CellMiner database suggested that SAAL1 expression is associated with chemoresistance to certain chemotherapeutic agents commonly used clinically. Lastly, the contribution of SAAL1 to tumorigenesis was suggested by our *in vitro* experiments, which showed that depletion of SAAL1 significantly inhibited proliferation, migration, and invasion, and reduced protein and mRNA expression of PD-L1 in lung cancer A549 cells. Overall, our findings suggest that SAAL1 not only contributes to the tumor onset and development but is also closely related to their response to chemotherapy and immunotherapy.

In summary, our study comprehensively analyzed the landscape of SAAL1 expression in different types of cancer. In general terms, our findings indicated that different mutations are associated with SAAL1 overexpression and pro-oncogenic activity in many solid tumors. Interestingly, our data indicate that SAAL1 may facilitate either resistance or sensitivity to chemotherapy and immunotherapies, depending on tumor type and expression levels. Our study has, however, some limitations. Most of our data were mined using public databases, and lack experimental verification. In this regard, and because lung cancer is by far the most prevalent tumor, we limited our *in vitro* studies addressing the tumorigenic role of SAAL1 to the A549 cell line, in which SAAL1 is overexpressed. Nevertheless, we expect our findings to be useful for guiding future research on the role of SAAL1 in other types of tumors and to help design novel strategies to improve cancer treatment.

## MATERIALS AND METHODS

### Expression analysis of SAAL1

SAAL1 expression in normal tissues was assessed via the Genotype-Tissue Expression database (GTEx; https://gtexportal.org) [23]. In addition, data downloaded from the Cancer Cell Line Encyclopedia (CCLE; https://sites.broadinstitute.org/ccle/) were used to analyze the expression of SAAL1 in cancer cell lines from 30 types of cancer. Then, RNA-seq datasets from 33 types of cancer in The Cancer Genome Atlas (TCGA; https://portal.gdc.cancer.gov/) [24] were used to analyze the differences in SAAL1 expression between tumor and paired normal tissue samples. Moreover, considering the small number or absence of normal samples for some tumor types in TCGA, normal tissue data from GTEx database were combined with the TCGA tumor tissue data to investigate the differential expression of SAAL1 in 33 cancerous tissues. The Human Protein Atlas database (HPA; https://www.proteinatlas.org/) was used to visually display the protein expression of SAAL1 in the form of immunohistochemical (IHC) staining. Furthermore, we used UALCAN portal (http://ualcan.path.uab.edu/index.html) [25] to conduct a protein expression analysis of SAAL1 between different tumors, using the Clinical Proteomic Tumor Analysis Consortium (CPTAC) dataset. In addition, the clinicopathological characteristics of the patients (smoking, age, and 8th World Health Organization [WHO] pathological stages) were obtained from TCGA and assessed for their association with SAAL1 expression. All data were obtained in January 2022.

### Survival analysis of SAAL1 in different types of cancer

Overall survival (OS) and progression-free interval (PFI) outcomes were obtained from the UCSC Xena database (https://xena.ucsc.edu/) and examined to assess the relationship between SAAL1 expression and patient prognosis. A univariate Cox model was used to evaluate the relationships between SAAL1 expression and various survival outcomes in a pan-cancer analysis. Data were visualized as forest plots using the “forestplot” (version 1.10.1) R package. The median of SAAL1 expression in each tumor was used as cutoff value to divide patients into high and low expression subgroups. The survival data of each cancer type were assessed by the Kaplan-Meier survival method, and survival curves were drawn using the R packages “survminer” (version 0.4.9) and “survival” (version 3.210). P<0.05 was considered significant.

### Analysis of SAAL1 gene alterations and promoter methylation status

The alteration status (alteration frequency and type) of the SAAL1 gene was analyzed in TCGA tumor datasets from cBioPortal (https://www.cbioportal.org/). This platform was further used to display mutated sites, using the Mutation-Mapper module, and to retrieve pan-cancer (with or without SAAL1 genetic alteration) OS data, using the Comparison module. Kaplan-Meier plots with Renyi-type tests and P-value were acquired as well. Data on the promoter methylation status of SAAL1 in LUAD and LUSC were obtained from the UALCAN database (http://ualcan.path.uab.edu/index.html). DNA methylation data were presented as β values ranging from 0 (unmethylated) to 1 (fully methylated).

### Correlation analysis of SAAL1 expression and immunological characteristics

The Cell type Identification by Estimating Relative Subsets of RNA Transcripts (CIBERSORT) portal (https://cibersort.stanford.edu) [26] was used to analyze the relationship between SAAL1 and 22 types of tumor-infiltrating immune cells in TCGA datasets, based on TME scores and gene sets related to three tumor microenvironment (TME)-relevant signatures: immune signatures (Antigen-processing-machinery, Immune-Checkpoint, CD8-T effector, TMEscoreA, and TMEscore), mismatch signatures (Mismatch-Repair, Nucleotide-excision-repair, DNA-damage-response, DNA-replication and Base-excision-repair), and stromal signatures (epithelial-to-mesenchymal transition (EMT) 1, EMT2, EMT3, pan tissue fibroblast TGF-β response signature (Pan-F-TBRs) and TMEscoreB). These signatures represent three core tumor immune biological pathways: (I) pre-existing T-cell immunity and (II) tumor mutation burden (TMB) is positively associated with immunotherapy outcome, whereas (III) TGF-β is associated with lack of immunotherapy response and reduced survival [27–29].

In addition, the relationship between SAAL1 expression and immune-related (e.g., immunostimulatory, immune checkpoint, major histocompatibility complex (MHC), chemokine and chemokine receptor) genes was examined using the TISIDB website (http://cis.hku.hk/TISIDB/) [30] through estimation of Pearson’s correlation coefficients.

### Functional enrichment analysis of SAAL1 in different types of cancer

To explore the involvement of SAAL1 in signal transduction pathways, gene set enrichment analysis (GSEA) [31] was performed on high and low SAAL1-expressing cancer types, using the median SAAL1 expression level as threshold. The top five terms for the gene sets in the Kyoto Encyclopedia of Genes and Genomes (KEGG) and Gene Ontology (GO) signatures were obtained from GSEA (https://www.gsea-msigdb.org/gsea/downloads.jsp). Pathways were considered significantly enriched based on NES>1, P<0.05, and FDR<0.25. The R packages ‘limma’ (version 3.44.3), ‘org.Hs.eg.db’ (version 3.11.4), ‘enrichplot’ (version 1.8.1), and ‘clusterProfiler’ (version 3.16.1) were applied for GSEA.

### Analysis of the association of SAAL1 with microsatellite instability (MSI), tumor mutational burden (TMB), and neoantigen load

MSI arises in tumors with deficient DNA mismatch repair and denotes hypermutability due to changes in genomic short tandem repeat sequences known as microsatellites [32]. TMB is defined as the total number of somatic mutations present in defined coding regions of the tumor genome [33]. Tumor neoantigens load arising from cancer-specific mutations generate a molecular fingerprint that has a definite specificity for cancer [34]. The genome-wide neoantigen landscape for each sample was predicted by NetMHCpan (version 3.0) [35]. MSI, TMB, and neoantigen load status impact the development of cancers and represent useful biomarkers for evaluating the therapeutic efficacy of immune checkpoint inhibitors. The analysis of the association between SAAL1 expression and MSI, TMB, and neoantigen load was carried out using Spearman’s correlation coefficient. The ‘fmsb’ (version 0.7.2), ‘limma’ (version 3.28.14), and ‘dplyr’ (version 0.7.8) R packages were used to analyze somatic data from different types of cancers in TCGA.

### Cohort validation of the prognostic effect of SAAL1 on immunotherapy

A systematic study of immune checkpoint gene expression profiles was performed by retrieving gene expression and immunotherapeutic effect data from the IMvigor210 cohort [27] using the ‘IMvigor210CoreBiologies’ package in R. Based on cohort data, the influence of SAAL expression (by RNA-Seq) on immunotherapy response (response; complete response (CR), partial response (PR); no response; progressive disease (PD); stable disease (SD)) was investigated using Chi-square tests. Furthermore, according to the correlation between SAAL1 expression and patient survival, the ‘surv-cutpoint’ function of the ‘survminer’ (version 0.4.9) R package was used to divide patients into high and low SAAL1 expression groups according to the median of the cohort. The Kaplan-Meier method and log-rank tests were used to analyze patient OS.

### Analysis of drug sensitivity of SAAL1

To evaluate the sensitivity of SAAL1 to anticancer drugs, Spearman’s correlation was applied to examine drug activity data and RNA-seq expression profiles from the NCI-60 panel of human cancer cell lines available in the CellMiner™ database (https://discover.nci.nih.gov/cellminer/home.do) [36]. Drugs approved by the FDA or used in clinical trials were selected for analysis, which was conducted using the ‘impute’ (version 1.46.0), ‘limma’ (version 3.28.14), ‘ggplot2’ (version 3.3.3), and ‘ggpubr’ (version 0.4.0) packages in R.

### Cell culture and small interfering RNA (siRNA) transfection

The human LUAD cell line A549 was purchased from the Cell Culture Center (Chinese Academy of Medical Sciences, Shanghai, China). The cell line was verified by STR profiling and was free of mycoplasma infection. A549 cells were cultured in 6-well plates in RPMI-1640 medium containing 10% fetal bovine serum (both from Hyclone|Cytiva, Logan, UT, USA) in a 37° C/5% CO_2_ incubator. When the cells reached 30%-50% confluence, SAAL1-targeting siRNAs, and corresponding control siRNAs (designed and synthesized by Shanghai GenePharma Co., Ltd., Shanghai, China) were introduced using Lipofectamine® 3000 reagent (Invitrogen; Thermo Fisher Scientific, Inc., Waltham, MA, USA) according to the manufacturer’s protocols. The transfected cells were incubated at 37° C and 5% CO_2_ for 48 h prior to experiments. The siRNAs sequences were: SAAL1-targeting siRNA1: sense: 5’-CCACCUACUCUGCUGGAAATT-3’; anti-sense: 5’-UUUCCAGCAGAGUAGGUGGTT-3’. SAAL1-targeting siRNA2: sense: 5’-GGUUGUGGACAAGCUCUUUTT-3’; anti-sense: 5’-AAAGAGCUUGUCCACAACCTT-3’. Control siRNA: sense: 5’-UUCUCCGAACGUGUCACGUTT-3’; anti-sense: 5’-ACGUGACACGUUCGGAGAATT-3’.

### Cell proliferation assays

Cell proliferation was evaluated using an EdU Cell Proliferation Assay kit (Cat. C0071S; Beyotime, Shanghai, China). Cells were seeded into 6-well plates at a density of 5×10^3^ cells/ml. Following incubation with 50 μM EdU solution for 12 h, cells were fixed in 4% paraformaldehyde for 30 min, and further incubated in 5% glycine for 5 min at room temperature. The cells were then washed in 1× PBS, followed by treatment with 0.5% Triton X-100 at room temperature for 30 min. Thereafter, 100 μl Apollo® mixture (Cat. C0071S; Beyotime) was added to each well for 30 min at room temperature. Cell nuclei were then stained using Hoechst 33342 solution (25° C, 25 min) and images were captured using a fluorescence microscope (Observer A1; ZEISS, Germany). The number of EdU-positive cells was counted using ImageJ software (version 1.8.0-172, National Institutes of Health).

Cell viability was evaluated using a CCK-8 kit (Beyotime Institute of Biotechnology). Transfected A549 cells were seeded in 96-well plates at a density of 5×10^3^ cells/well and viability examined at 6, 24, 48, 72, and 96 h post-transfection after 2-h incubation in CCK-8 solution, according to the manufacturer’s protocol. Absorbance was measured at 450 nm using a microplate reader (Tecan Infinite M200PRO; Tecan Group Ltd).

### Transwell migration and invasion assays

Transwell assays were done as reported in the previous study [37]. For cell invasion assays, Matrigel (Corning, NY, USA) was diluted at a 1:3 ratio and spread evenly onto the bottom of 24-well transwell inserts. Uncoated inserts were used for migration assays. Non-transfected A549 cells, as well as control siRNA-, siRNA1-, and siRNA2-transfected A549 cells were harvested and counted during the logarithmic growth phase, and a cell suspension (1×10^6^ cells/ml) was prepared with serum-free RPMI 1640 medium. Cell suspension (150 μl) was added to each chamber in a 24-well transwell plate (Corning, NY, USA), and 600 μl RPMI 1640 medium containing 20% FBS was added to each lower chamber. Subsequently, the upper chambers were inserted into the lower chambers and placed in a 5% CO_2_ incubator at 37° C for 24 h. The inserts were then removed and fixed in 4% paraformaldehyde prior to crystal violet staining. Cells were counted using ImageJ software in randomly selected visual fields at 100× magnification using a fluorescence microscope (Observer A1; ZEISS).

### Western blotting

Cells were lysed using lysis buffer (Cat. 9803S; Cell Signaling Technology Inc., Danvers, MA, USA) supplemented with a protease inhibitor cocktail (Cat. 11697498001; Roche Diagnostics, GmbH) for 30 min at 4° C. Protein concentration was measured by bicinchoninic acid (BCA) assay (Thermo Fisher Scientific, Inc.). A total of 30 μg of protein per lane were separated using 10% sodium dodecyl sulfate-polyacrylamide gel electrophoresis (SDS-PAGE) (Bio-Rad Laboratories, Inc., Hercules, CA, USA) and transferred to PVDF membranes (Merck Life Sciences, Inc., Australia). After blocking for 2 h with 5% skimmed dried milk, the membranes were washed with 1×TBST (1×Tris-Buffered Saline, 0.1% Tween 20) and incubated at 4° C overnight with rabbit polyclonal primary antibodies (1:1000 dilution; Novus Biologicals Inc., UK) against SAAL1 (Cat. NBP1-83447), PD-L1 (Cat. NBP1-76769), and GAPDH (Cat. NB300-327). After 1×TBST wash, the membranes were incubated with an anti-rabbit IgG secondary antibody (Cat. NBP1-75293; 1:3000 dilution; Novus Biologicals, Inc.) at room temperature for 2 h. Immunoreactive bands were detected using an ECL western blotting system (Clarity Western ECL Substrate; Bio-Rad Laboratories, Inc.). Band densities were measured using ImageJ software, and normalized to those of GAPDH.

### Quantitative reverse transcription PCR (RT-qPCR) analysis

Total RNA was extracted from A549 cells 48 h after transfection using TRIzol (Invitrogen; Thermo Fisher Scientific, Inc.). Reverse transcription was performed using a PrimeScript RT Kit with gDNA Eraser (Cat. RR047A; Takara Bio, Inc., Japan) according to the manufacturer’s protocol. RT-qPCR was done using SYBR Green reagent (Takara Bio, Inc.) as reported in our previous study [38]. Relative SAAL1 and PD-L1 expression was analyzed using the 2^-ΔΔCq^ method, with GAPDH as internal reference. The primer sequences were: SAAL1, 5’-GGAGTACTGGCCAAGTCCAAGTG-3’ (forward) and 5’-CCAGCAGAGTAGGTGGGTCTGAA-3’ (reverse); PD-L1, 5’-GTGGCATCCAAGATACAAACTCAA-3’ (forward) and 5’-TCCTTCCTCTTGTCACGCTCA-3’ (reverse); and GAPDH, 5’-ATGTTCCAGTATGACTCCACTCACG-3’ (forward) and 5’-GAAGACACCAGTAGACTCCACGACA-3’ (reverse).

### Statistical analysis

All statistical analyses were performed using R statistical software (version 4.0.3). In bioinformatics analyses, Kruskal-Wallis tests were performed to examine differences in SAAL1 expression between different tissue types and cancer cell lines. The significance of the difference in gene expression between cancerous and para-cancerous normal tissues was determined using Wilcoxon’s tests. Patient prognosis was evaluated using univariate Cox regression, and results are presented as hazard ratio (HR), 95% confidence interval (CI), and P-values. The KM method with log-rank tests was used to estimate survival probability against time. When late-stage crossover was observed between groups, Renyi-type tests were applied and results presented as P-values. The correlation between SAAL1 expression and immunological characteristics was evaluated using Pearson’s or Spearman’s correlation tests. P<0.05 indicated a statistically significant difference.

All data obtained from *in vitro* experiments were expressed as the mean ± SD. Comparisons between groups were performed using one-way ANOVA (CCK-8 assay, EdU assay, western blotting, and RT-qPCR) followed by Tukey’s post hoc tests. P<0.05 was considered significant. All experiments were performed in triplicate.

### Availability of data and materials

The datasets used and/or analyzed during the current study are available from the corresponding author on reasonable request.
